# Low Testosterone on Social Media: Application of Natural Language Processing to Understand Patients’ Perceptions of Hypogonadism and Its Treatment

**DOI:** 10.2196/21383

**Published:** 2020-10-07

**Authors:** Vadim Osadchiy, Tommy Jiang, Jesse Nelson Mills, Sriram Venkata Eleswarapu

**Affiliations:** 1 Division of Andrology Department of Urology David Geffen School of Medicine, University of California, Los Angeles Los Angeles, CA United States; 2 Consortium for Health Activity on Social Media David Geffen School of Medicine University of California, Los Angeles Los Angeles, CA United States

**Keywords:** hypogonadism, natural language processing, Reddit, social media, testosterone replacement therapy, Twitter

## Abstract

**Background:**

Despite the results of the Testosterone Trials, physicians remain uncomfortable treating men with hypogonadism. Discouraged, men increasingly turn to social media to discuss medical concerns.

**Objective:**

The goal of the research was to apply natural language processing (NLP) techniques to social media posts for identification of themes of discussion regarding low testosterone and testosterone replacement therapy (TRT) in order to inform how physicians may better evaluate and counsel patients.

**Methods:**

We retrospectively extracted posts from the Reddit community r/Testosterone from December 2015 through May 2019. We applied an NLP technique called the meaning extraction method with principal component analysis (MEM/PCA) to computationally derive discussion themes. We then performed a prospective analysis of Twitter data (tweets) that contained the terms low testosterone, low T, and testosterone replacement from June through September 2019.

**Results:**

A total of 199,335 Reddit posts and 6659 tweets were analyzed. MEM/PCA revealed dominant themes of discussion: symptoms of hypogonadism, seeing a doctor, results of laboratory tests, derogatory comments and insults, TRT medications, and cardiovascular risk. More than 25% of Reddit posts contained the term doctor, and more than 5% urologist.

**Conclusions:**

This study represents the first NLP evaluation of the social media landscape surrounding hypogonadism and TRT. Although physicians traditionally limit their practices to within their clinic walls, the ubiquity of social media demands that physicians understand what patients discuss online. Physicians may do well to bring up online discussions during clinic consultations for low testosterone to pull back the curtain and dispel myths.

## Introduction

The Testosterone Trials were a coordinated series of placebo-controlled, double-blinded trials intended to elucidate risks and benefits of testosterone replacement therapy (TRT) in hypogonadal men [1-7]. Despite these recent trials, clinicians continue to be uncomfortable treating these men, in part due to unanswered questions related to cardiovascular outcomes and cancer risk, as well as how TRT is portrayed in popular culture. Perhaps discouraged by conflicting information from physicians and traditional media, patients sometimes turn to social media platforms to discuss medical concerns with peers [8,9].

Interactive social media channels have emerged as potent resources for individuals to discuss health care concerns [9]. Reddit, an anonymous discussion platform with over 330 million monthly active users, serves as a popular internet destination for discussions of health-related topics [10]. The Reddit forum or subreddit r/Testosterone [11], which boasts over 30,000 active members, is devoted to answering questions, sharing personal accounts, and disseminating resources related to TRT and testosterone levels. Similar discussions occur on other social media sites, including Twitter, a microblogging platform with over 126 million daily active users [12].

We hypothesized that the content of online discussions about low testosterone can be classified into themes that may inform how physicians evaluate, counsel, and treat men with hypogonadism. Here, we apply quantitative natural language processing (NLP) techniques to identify dominant themes of discussions regarding low testosterone and TRT on social media.

## Methods

### Study Design and Sources of Data

**Figure 1:**
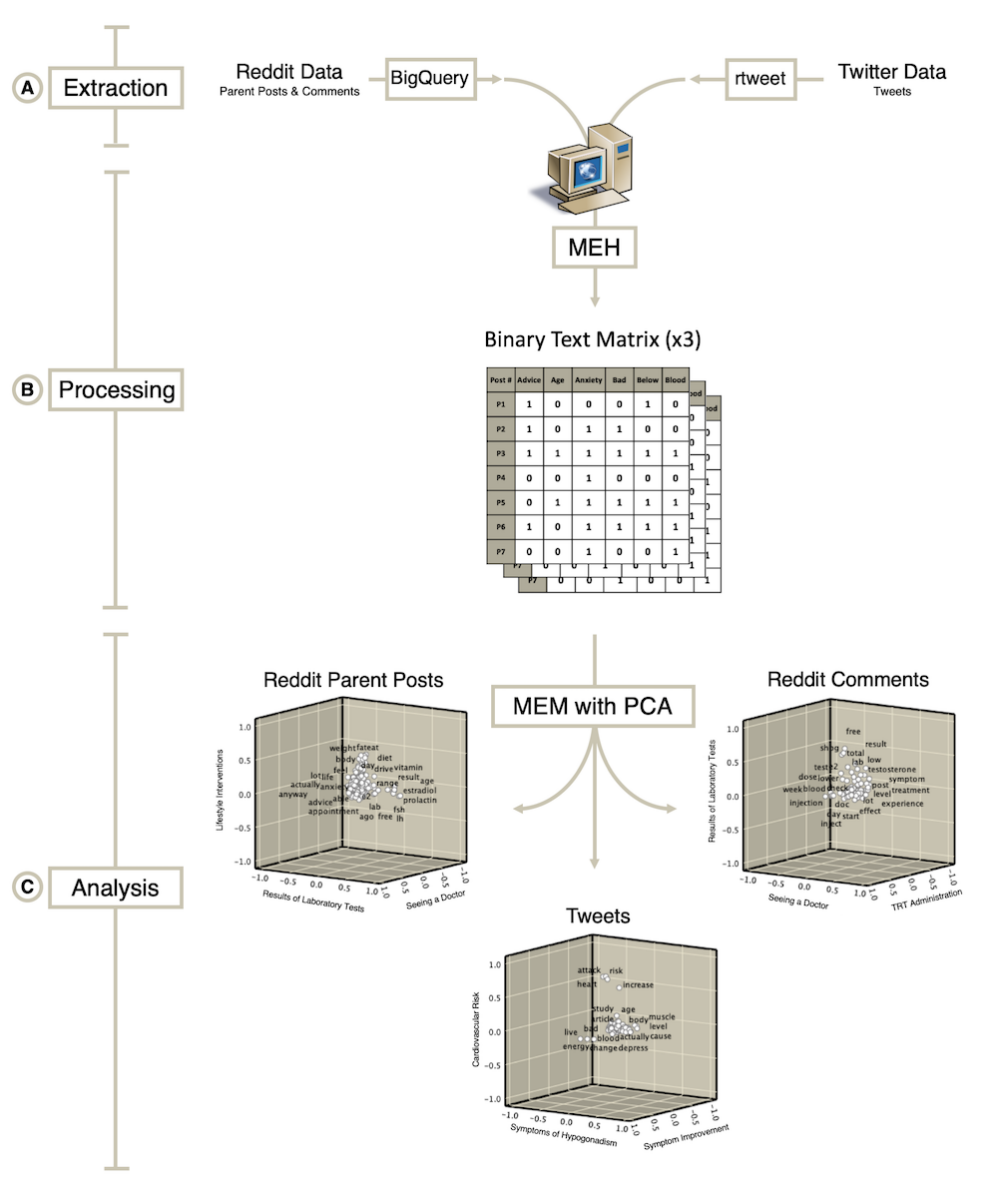
Overview of methods: (A) extraction of Reddit and Twitter data using BigQuery and rtweet, respectively; (B) processing of raw text data using the meaning extraction helper to generate a binary text matrix for each data set; (C) meaning extraction method with principal component analysis generates word clusters for each dataset. Rotated component plots are shown with x-, y-, and z-axes representing the three clusters that capture the greatest variance of the data. MEH: meaning extraction helper; MEM: meaning extraction method; PCA: principal component analysis.

An overview of our methodology is presented in Figure 1. The study comprised three phases: extraction of data from social media platforms (Figure 1A), automated organization of textual data (Figure 1B), and quantitative analysis of the textual data to identify dominant themes of the text (Figure 1C).

First, we retrospectively processed posts and comments from the Reddit community r/Testosterone from December 2015 through May 2019. Reddit data were extracted using BigQuery (Google LLC), an enterprise data analytics platform, from a dataset uploaded for public use [13] (Figure 1A). We evaluated both parent posts (the main post in a Reddit discussion) and comment posts (submitted in response to a parent post). We applied a word count criterion of >20 words for parent posts to exclude potential spam, deleted text, and posts composed only of links to other websites. As we anticipated the average word count of comment posts to be less, we used a more relaxed word count criterion of >5 words for comment posts.

Next, Twitter data (tweets) were collected prospectively from June through September 2019 using the rtweet application [14], which integrates tweets for processing in RStudio version 1.1.463 (RStudio PBC) (Figure 1A). We extracted tweets containing the terms low testosterone, low T, and testosterone replacement. We applied a word count criterion for tweets (>5 words per tweet), given the character count limitation imposed by the Twitter platform. Retweets (reposts of an identical, previously published tweet) were excluded from analysis.

### Natural Language Processing Using the Meaning Extraction Method

Reddit parent posts, Reddit comment posts, and tweets from Twitter were separately subjected to an NLP technique called the meaning extraction method (MEM) [15] with principal component analysis (PCA). MEM/PCA tracks words that cluster together to derive themes quantitatively [15]. This approach has been previously validated to reveal information about individuals’ personalities, communication strategies, and behaviors [16,17].

To automate the MEM, we used the topic modeling application meaning extraction helper version 2 [18] to deconstruct each post or tweet into its component words. Stop words (eg, articles, prepositions, and transitions) were filtered out. Remaining words were ranked by their frequencies of appearance in each post or tweet (Figure 1B). Words were then subjected to PCA with varimax rotation (Figure 1C) using SPSS Statistics version 25 (IBM Corporation). PCA identified clusters of words that frequently appeared together. Each word was conferred a factor loading, the correlation coefficient between the word and the cluster to which it belonged. Factor loading thresholds of >0.20 are appropriate when performing PCA of text data to capture a sufficient proportion of the variance in the data [19,20]. We assigned a descriptive theme to each cluster based on the words within it.

### Subset Analyses on Key Topics of Interest

Given widespread interest and controversy regarding the potential associations of TRT with cardiovascular disease and prostate cancer risk, we sought to quantitate the appearance of these topics on Reddit and Twitter. Subset analysis was performed to determine the frequencies of the words prostate, cancer, PSA (prostate-specific antigen), heart, attack, stroke, cardiovascular, and death. Furthermore, to identify the degree to which individuals allude to seeking consultation with a health care provider, an additional analysis was performed to determine the frequencies of the relevant terms doctor, urologist, endocrinologist, and appointment.

### Statistical Validity of Principal Component Analysis

To assess applicability of PCA to each dataset, the Kaiser-Meyer-Olkin (KMO) statistic, a measure of sampling adequacy (values >0.60 are adequate), and the Bartlett test for sphericity, which tests if there are significant correlations among variables of interest, were calculated [21].

### Ethics

Consistent with previous investigations on social media data, this work was exempted by the institutional review board of the University of California, Los Angeles, as it involves publicly available data and does not involve human subjects.

## Results

### Total Number of Posts Extracted From Social Media

From the r/Testosterone community on Reddit, we retrospectively extracted 19,083 parent posts and 218,082 comment posts over the 42-month period of study. After exclusions, 12,665 parent posts and 186,670 comment posts remained. From Twitter, we prospectively extracted 7467 tweets over 4 months; 6659 tweets remained after exclusions.

### Natural Language Processing of Reddit Data

Using MEM for Reddit parent post and comment post data, we identified 5 factors, or thematic word clusters, that included words with factor loadings greater than 0.30 and 0.20, respectively (Tables 1 and 2).

The following themes emerged from NLP of Reddit data: seeing a doctor, results of laboratory tests, administration of TRT, and lifestyle interventions (both parent posts and comment posts); symptoms of hypogonadism (parent posts only); and TRT medications (comment posts only). Table 3 contains representative quotations that feature each Reddit theme. Some quotes have been abridged in the interest of space.

**Table 1:** Thematic clusters, word frequencies, and associated factor loading coefficients derived from the meaning extraction method with principal component analysis of parent posts from the Reddit community r/Testosterone (n=12,665).

Cluster and word		Factor loading coefficient	Frequency
**Results of laboratory tests**			
	LH (luteinizing hormone)	0.72	13.2
	FSH (follicle-stimulating hormone)	0.70	11.4
	Free	0.67	26.3
	Prolactin	0.61	8.8
	TSH (thyroid-stimulating hormone)	0.58	7.7
	SHBG (sex hormone binding globulin)	0.57	12.3
	Total	0.56	24.3
	Estradiol	0.54	11.3
	Range	0.46	22.1
	Result	0.41	26.0
**Lifestyle interventions**			
	Weight	0.55	10.6
	Fat	0.55	9.3
	Eat	0.53	8.4
	Diet	0.50	8.7
	Gain	0.49	7.3
	Lift	0.48	7.0
	Muscle	0.46	9.8
	Sleep	0.43	11.3
	Gym	0.40	6.7
	Body	0.38	11.6
**Seeing a doctor**			
	Doctor	0.43	25.5
	Low	0.37	43.3
	Told	0.37	8.4
	Level	0.35	34.6
	Month	0.32	26.4
	Appointment	0.32	5.1
	Read	0.31	13.1
	Endocrinologist	0.31	5.2
	Treatment	0.31	7.5
	Urologist	0.30	5.7
**Testosterone replacement therapy administration**			
	Week	0.55	40.2
	Dose	0.49	13.3
	HCG (human chorionic gonadotropin)	0.46	13.5
	Protocol	0.42	6.3
	Injection	0.42	16.4
	Day	0.42	28.4
	CYP (cytochrome P450)	0.42	5.9
	Start	0.40	28.7
	E2 (estradiol)	0.38	12.3
	Twice	0.36	5.8
**Symptoms of hypogonadism**			
	Fog	0.76	5.0
	Brain	0.75	5.7
	Depress	0.42	11.7
	Symptom	0.40	21.0
	Anxiety	0.38	7.9
	Libido	0.36	16.0
	Erection	0.35	8.4
	Sex	0.34	14.7
	Energy	0.32	10.7
	Drive	0.31	9.1

**Table 2:** Thematic clusters, word frequencies, and associated factor loading coefficients derived from the meaning extraction method with principal component analysis of comment posts from the Reddit community r/Testosterone (n=186,670).

Cluster and word		Factor loading coefficient	Frequency
**Seeing a doctor**			
	Doctor	0.39	9.0
	TRT (testosterone replacement therapy)	0.38	15.3
	Treatment	0.33	2.7
	Symptom	0.32	5.9
	Life	0.31	4.0
	People	0.30	5.9
	Issue	0.29	5.3
	Help	0.27	6.0
	Cause	0.27	5.6
	Hormone	0.26	3.9
	Prescribe	0.26	2.6
	Experience	0.25	2.8
**Results of laboratory tests**			
	Free	0.64	4.9
	Total	0.58	4.9
	SHBG (sex hormone binding globulin)	0.54	4.6
	Range	0.44	5.9
	Test	0.42	17.9
	Low	0.38	15.4
	LH (luteinizing hormone)	0.34	2.8
	Lab	0.33	4.5
	E2 (estradiol)	0.31	8.0
	Normal	0.28	5.6
	Result	0.26	4.3
**Testosterone replacement therapy administration**			
	Week	0.60	13.0
	Day	0.45	9.7
	Injection	0.43	5.8
	Dose	0.42	7.6
	Inject	0.35	3.9
	Start	0.33	9.1
	Protocol	0.32	2.8
	Feel	0.31	11.6
	Month	0.31	7.2
	Time	0.30	9.4
**Lifestyle interventions**			
	Fat	0.57	3.0
	Eat	0.55	3.1
	Diet	0.53	3.4
	Weight	0.50	3.2
	Muscle	0.41	2.8
	Body	0.34	4.7
	Sleep	0.33	3.5
**Testosterone replacement therapy medications**			
	Increase	0.40	4.2
	Estrogen	0.37	3.3
	Effect	0.36	4.2
	Lower	0.32	4.7
	Testosterone	0.30	11.9
	Clomid	0.26	3.7
	HCG (human chorionic gonadotropin)	0.26	5.9

**Table 3:** Representative quotations for each theme derived from the meaning extraction method. Asterisks are part of the quotations and do not refer to anything in the table.

Data source and theme		Representative quotation
**Reddit parent posts**		
	**Results of laboratory tests**	
		Here’s what came up: FSH^a^ 2.1 (1.5-12.4) LH^b^ 5.6 (1.7-8.6) Prolactin 15.25 (4.04-15.2)* T, total^c^ 311.1 (249-836)* SHBG^d^ 33.3 (16.5-55.9) Free testosterone index 32.43 (35.0-92.6)*
		shbg and dhea still pending. I had to get these results because i have an appointment with neurosurgeon soon and he will need the labs and mri^e^.
	**Lifestyle interventions**	
		Have been eating super clean. Working with a dietitian/personal trainer. Was dieting mostly high protein / low fat / low carb
		I work out all the time lifting heavy weights, 3 or 4 times a week on average. I eat a good diet, take my zinc, vitamin D, and get in my fats and essential fats.
	**Seeing a doctor**	
		I know several people on trt^f^, but they all have the same doc...you walk in, tell him you want to get bigger, stronger, and faster, pay out of pocket for his blood test then buy your meds from his attached pharmacy. That’s not what I want. I want to find out what’s wrong without a preconceived bias.
		So I go to the appointment. And the specialist I saw (a urologist) said he wasn’t the guy to see about this issue, and ended up referring me to another specialist.
	**Testosterone replacement therapy administration**	
		T cyp^g^ 200 mg/ml - 0.32 mL IM/SQ twice weekly (~130 mg/week) HCG^h^ 500 IU SQ twice weekly to prevent testicular atrophy No AI^i^ - low E2^j^, monitor DHEA^k^ 25 mg every night
		So I don’t know what to do? Take my AI and hope that my E2 is high? Or keep not taking my AI and hope things will get better? I literally can’t hold out a week to get another blood test and also I can’t afford it right now.
	**Symptoms of hypogonadism**	
		All the normal symptoms: brain fog, mood swings, low libido, erectile dysfunction, inability to add muscle at the gym despite working out 3x a week.
		Symptoms: brain fog, very low energy level, lifelessness-zombie feeling most days, very lethargic, mood swings, easy to get angry, grumpy and annoyed at earliest, no libido/sex drive, ED^l^—less frequency, less powerful, minimal to no erections during sex, softer (haven’t had sex in years)
**Reddit comment posts**		
	**Seeing a doctor**	
		Many doctors—especially PCPs^m^—are not fluent in the endocrine system. They aren’t supposed to be. Going to your primary care physician for hormone questions is a mistake. If you knew you had heart issues, wouldn’t you go to a cardiologist?
		My PCP looked super confused and clueless as to what he was supposed to do for me. Doc made me do two more labs fasting to confirm then he referred me out to an endocrinologist. The endo made me do three more fasting labs and a testicular ultrasound to confirm.
	**Results of laboratory tests**	
		Honestly I don’t think testosterone is your problem based on Sept 10th 2015 blood results. You have decent midrange total, and free testosterone. SHBG bounces around, so maybe it’s a testing error.
		198 is low as hell for your dad, and even 450 for him would be low. Yours is lowish, but you have definite symptoms.
	**Testosterone replacement therapy administration**	
		75 mg E5D^n^ (105 mg per week). Doesn’t require an AI, doesn’t give me side effects. I am at ~700 on trough days and feel pretty damn good.
		I had just moved to a standard TRT dose of Test Cyp, 100 mg/week. At 5’11”, 172 lbs, and 17% body fat, taking 1 mg of Arimidex every day tanked my E2. Dropping down to 0.25 mg Arimidex once a week had the same effect.
	**Lifestyle interventions**	
		Eat good food, lift heavy, and get sleep. Repeat for two years.
		TRT will not turn you into a bodybuilder. It may tone you a little bit (if everything is in check). But just saying “I eat good” literally means nothing. What are your macros? What’s your diet? Etc?
	**Testosterone replacement therapy medications**	
		HCG is a water-based peptide hormone that can be injected to replace the lost LH hormone that TRT shuts down. Without hCG, the LH receptors in the testes are no longer getting activated. The results: the testes shrink.
		Clomiphene. What a double-edged sword. First, Clomid will certainly have an effect on your testosterone levels. Usually, it is doses substantially higher than 12.5 mgs daily.
**Tweets**		
	**Symptoms of hypogonadism**	
		Keeping your hormone levels up is a crucial part of #health. Low T can lead to all types of adverse effects: - weight gain/belly fat - #LowEnergy - low sex drive
		This, in turn, causes a lower sex drive, depression, reduced muscle mass, and low levels of energy. Erectile dysfunction is another symptom.
	**Cardiovascular risk**	
		#Testosterone Replacement Therapy Lowers Heart Attack Risk
		Aging men with low testosterone levels who take testosterone replacement therapy (TRT) are at a slightly greater risk of experiencing an ischemic stroke
	**Symptom improvement**	
		Starting testosterone replacement therapy and thyroid medication at the same time is quite the 1-2 punch to the system. Endless energy, great sleep, and able to lift weights heavier and longer.
		“My energy is back”: how testosterone replacement therapy is changing men’s lives
	**Derogatory comments and insults**	
		That little cuck should be the poster boy for low T supplements
		I was called effeminate and a low testosterone beta here for defending women’s rights.

^a^FSH: follicle-stimulating hormone.

^b^LH: luteinizing hormone.

^c^T, total: total testosterone.

^d^SHBG: sex hormone binding globulin.

^e^MRI: magnetic resonance imaging.

^f^TRT: testosterone replacement therapy.

^g^CYP: cytochrome P450.

^h^HCG: human chorionic gonadotropin.

^i^AI: aromatase inhibitor.

^j^E2: estradiol.

^k^DHEA: dehydroepiandrosterone.

^l^ED: erectile dysfunction.

^m^PCP: primary care physician.

^n^E5D: every 5 days.

The highest frequency word occurrences among parent posts as determined by PCA were low (5484/12,665 [43.30%] of posts), week (5092/12,665, 40.20%), level (4382/12,665, 34.60%), and start (3635/12,665, 28.70%). Among comment posts, the highest frequency word occurrences were test (33,414/186,670, 17.90%), low (28,747/186,670, 15.40%), TRT (28,561/186,670, 15.30%), and week (24,267/186,670, 13.00%).

Parent post and comment post PCA accounted for 15.45% (1957/12,665) and 13.84% (25,835/186,670) of the total variance, respectively. KMO statistic was 0.91 for Reddit parent post data and 0.80 for Reddit comment post data, with Bartlett test <0.01, indicating that the Reddit data were appropriate for factor analysis using PCA.

### Natural Language Processing of Twitter Data

Similarly, MEM for Twitter data identified 4 factors, or thematic word clusters, with factor loadings greater than 0.25 (Table 4). The following themes emerged from NLP of tweets: symptoms of hypogonadism, cardiovascular risk, symptom improvement, and derogatory comments and insults.

The highest frequency word occurrences among tweets as determined by PCA were level (693/6659, 10.40%), male (426/6659, 6.40%), sex (213/6659, 3.20%), and increase (200/6659, 3.00%). Twitter PCA accounted for 9.01% (600/6659) of the total variance. KMO statistic was 0.61 for Twitter data, with Bartlett test <0.01, indicating that the Twitter data were appropriate for factor analysis using PCA. Of note, other studies using MEM/PCA have reported similar percentages of variance as those determined in our analysis of Reddit and Twitter data [22,23].

**Table 4:** Thematic clusters, word frequencies, and associated factor loading coefficients derived from the meaning extraction method with principal component analysis of tweets about low testosterone, low T, or testosterone replacement on Twitter (n=6659).

Cluster and word		Factor loading coefficient		Frequency	
**Symptoms of hypogonadism**					
	Muscle		0.54		1.9
	Mass		0.48		1.2
	Sex		0.41		3.2
	Libido		0.39		1.0
	Level		0.36		10.4
	Drive		0.35		1.8
	Fat		0.32		1.3
	Hormone		0.31		2.7
	Body		0.28		2.3
	Weight		0.27		1.1
**Cardiovascular risk**					
	Heart		0.76		1.1
	Attack		0.76		1.1
	Risk		0.73		2.1
	Increase		0.62		3.0
**Symptom improvement**					
	Change		0.69		2.2
	Energy		0.69		2.9
	Live		0.69		2.0
	Life		0.21		2.1
**Derogatory comments and insults**					
	Boy		0.58		2.3
	Soy		0.56		2.4
	Beta		0.42		2.8
	Cuck		0.29		1.0
	Male		0.23		6.4
	Girl		0.22		1.0
	Little		0.22		1.3

### Word Occurrences on Key Topics of Interest

Subset analysis was performed to determine word occurrence frequencies in three key topics of interest that relate to TRT: prostate cancer risk, cardiovascular disease risk, and seeking consultation with a health care professional. These data are presented in Table 5.

In brief, over 1% of Reddit parent posts contain the terms prostate (143/12,665, 1.13%), cancer (143/12,665, 1.13%), PSA (210/12,665, 1.66%), or heart (175/12,665, 1.38%). Over a quarter of Reddit parent posts contain the term doctor (3235/12,665, 25.54%), while over 5% of parent posts refer to either a urologist (732/12,665, 5.78%) or endocrinologist (657/12,665, 5.19%). Frequencies of these terms were higher among Reddit posts than among tweets from Twitter.

**Table 5:** Subset analysis of word frequencies related to prostate cancer risk, cardiovascular disease risk, and seeking a health care consultation.

Concern associated with testosterone replacement therapy		Word frequency (%)		
		Reddit parent post	Reddit comment post	Tweet
**Prostate cancer risk**				
	Prostate	1.13	0.36	0.63
	Cancer	1.13	0.56	0.87
	PSA^a^	1.66	0.25	0.06
**Cardiovascular disease risk**				
	Heart	1.38	0.61	1.14
	Attack	0.89	0.32	1.14
	Stroke	0.23	0.13	0.86
	Cardiovascular	0.13	0.13	0.44
	Death	0.24	0.19	0.21
**Seeking health care consultation**				
	Doctor	25.54	9.00	1.77
	Urologist	5.78	1.36	0.20
	Endocrinologist	5.19	0.97	0.17
	Appointment	5.13	0.74	0.39

^a^PSA: prostate-specific antigen.

## Discussion

### Principal Findings

NLP techniques applied to unfiltered discussions on Reddit and Twitter offer a useful framework for understanding patient priorities outside the doctor’s office. We found that men largely turn to social media to learn about symptoms of low testosterone, interpretation of personal lab results, practicalities of TRT, and body changes with treatment. Notably, cardiovascular risk was a major discussion theme, echoing concerns among prescribers, who may be deterred by continued ambiguity despite the publication of the Testosterone Trials. Although NLP analysis did not reveal prostate cancer as a notable theme, a number of posts included text related to this topic, suggesting that this may represent an important discussion point for a subset of online discussions related to TRT.

Our results underscore that patients are searching for medical guidance related to hypogonadism on social media, an environment where anecdotes predominate and advertising often masquerades as medical advice [24]. TRT prescriptions have risen almost 4-fold over the last two decades, which can be attributed, in part, to off-label indications and direct-to-consumer advertising [25]. Even beyond standard TRT, testosterone-boosting supplements with minimal data to support their efficacy are aggressively marketed and readily available online [26]. But still, social media represents an enormous opportunity for the medical community to improve how we engage with our patients and to do so in a meaningful and impactful way. Potential interventions that may inoculate against coercive direct-to-consumer marketing practices include disseminating high-quality, open-access information related to hypogonadism. For example, Halpern et al [27] recently published a JAMA Patient Page article on hypogonadism. This single-page handout written in easily accessible language includes an infographic highlighting symptoms of hypogonadism and potential adverse effects of TRT, in addition to information related to etiology of hypogonadism and a discussion of potential cardiovascular and prostate cancer risks associated with TRT—all topics that emerged as major themes of discussion from our data.

Social media platforms, including Reddit and Twitter, create a space for patients not only to obtain answers to questions that they are either uncomfortable or unwilling to ask in a face-to-face clinical setting but also to connect with others going through similar experiences. However, not all health-related discussions online are productive. Twitter featured the theme of derogatory comments and insults, highlighting an undertone of stigma, which may compound existing barriers preventing men from accessing care [28]. In contrast, the seeing a doctor theme only emerged on Reddit, with more than 25% of parent posts mentioning the word doctor, compared with less than 2% on Twitter. This may reflect inherent differences among the two social media platforms, as Twitter is constrained by a strict character count limitation and is overall less anonymous, with discussants frequently using their true identities in their display usernames and account photos.

Although clinician engagement with the online hypogonadism community will become increasingly important in the coming years, improving the in-office clinical experience of our patients cannot be overemphasized. Our data reveal that many of the online discussions featured personal questions related to interpretation of lab results. This is consistent with a previous study exploring Reddit discussions of male factor infertility, where nearly 20% of all posts featured a question related to personal semen analysis results [29]. Such discussions related to lab results cannot be addressed by disseminating a primer on hypogonadism and TRT, but instead demand the expertise of a clinician trained in managing male endocrinology and the related sexual, reproductive, and psychological comorbidities. Creating an in-office experience where men feel comfortable and safe to ask their questions and voice their concerns should be a priority for any outpatient clinical setting, but especially one that caters to men with suspected hypogonadism. Both outpatient primary care settings and urological outpatient clinics can learn from the success of the emerging multidisciplinary men’s health clinic [30].

Here we offer valuable insight into primarily patient concerns in a forum that allows for honest and unfiltered patient feedback as it relates to these discussants’ experiences with hypogonadism. Clinically, these data highlight that patients worry most about comorbidities, lifestyle factors impacted by low testosterone, and treatment options. While other aspects of hypogonadism can be discussed, these data highlight the most salient hypogonadism-related concerns for our patients. Additionally, this study can further improve on patients’ in-office experiences by informing how physicians can lead discussions to highlight aspects of low testosterone that patients may feel are not being adequately addressed.

### Limitations

Our study is not without limitations. Although NLP techniques allowed us to analyze a large volume of discrete social media posts, generalizability of MEM is limited by the absence of contextual valence (positivity or negativity). However, this does not impair overall thematic identification. Additionally, discussants who turn to social media for health care information may be different with respect to demographics, health care priorities, and information preferences compared with those who do not; our results should therefore be interpreted within this context [31]. It should also be noted that some individuals use social media as a platform to vent about their experiences with health care professionals as they relate to hypogonadism care. This is an important distinction to make because it may not necessarily represent a lack of communication between patients and their physician but rather a discussant’s opportunity to share. Future studies may consider investigating to other Reddit communities, expanding Twitter search terms, or exploring other social media platforms.

### Conclusions

This study represents the first evaluation of the social media landscape surrounding hypogonadism and TRT using NLP techniques. Our analysis of more than 200,000 discrete social media posts revealed dominant themes of discussion, which may inform how physicians evaluate and counsel men with hypogonadism. Understanding the complex internet landscape of hypogonadism discussions represents the first step in creating well-informed and clinically meaningful change. Although physicians traditionally limit their practices to within their clinic walls, the ubiquity of social media demands that physicians engage patients where they are, including online. Practicing physicians may do well to bring up online discussions during clinic consultations, to pull back the curtain and dispel myths.

## Abbreviations

KMO: Kaiser-Meyer-Olkin statistic
MEM: meaning extraction method
NLP: natural language processing
PCA: principal component analysis
PSA: prostate-specific antigen
TRT: testosterone replacement therapy

## References

[1] Snyder PJ, Bhasin S, Cunningham GR, Matsumoto AM, Stephens-Shields AJ, Cauley JA, Gill TM, Barrett-Connor E, Swerdloff RS, Wang C, Ensrud KE, Lewis CE, Farrar JT, Cella D, Rosen RC, Pahor M, Crandall JP, Molitch ME, Resnick SM, Budoff M, Mohler ER, Wenger NK, Cohen HJ, Schrier S, Keaveny TM, Kopperdahl D, Lee D, Cifelli D, Ellenberg SS (2018). Lessons from the Testosterone Trials. Endocr Rev.

[2] Bhasin S, Ellenberg SS, Storer TW, Basaria S, Pahor M, Stephens-Shields AJ, Cauley JA, Ensrud KE, Farrar JT, Cella D, Matsumoto AM, Cunningham GR, Swerdloff RS, Wang C, Lewis CE, Molitch ME, Barrett-Connor E, Crandall JP, Hou X, Preston P, Cifelli D, Snyder PJ, Gill TM (2018). Effect of testosterone replacement on measures of mobility in older men with mobility limitation and low testosterone concentrations: secondary analyses of the Testosterone Trials. Lancet Diabetes Endocrinol.

[3] Mohler ER, Ellenberg SS, Lewis CE, Wenger NK, Budoff MJ, Lewis MR, Barrett-Connor E, Swerdloff RS, Stephens-Shields A, Bhasin S, Cauley JA, Crandall JP, Cunningham GR, Ensrud KE, Gill TM, Matsumoto AM, Molitch ME, Pahor M, Preston PE, Hou X, Cifelli D, Snyder PJ (2018). The effect of testosterone on cardiovascular biomarkers in the Testosterone Trials. J Clin Endocrinol Metab.

[4] Resnick SM, Matsumoto AM, Stephens-Shields AJ, Ellenberg SS, Gill TM, Shumaker SA, Pleasants DD, Barrett-Connor E, Bhasin S, Cauley JA, Cella D, Crandall JP, Cunningham GR, Ensrud KE, Farrar JT, Lewis CE, Molitch ME, Pahor M, Swerdloff RS, Cifelli D, Anton S, Basaria S, Diem SJ, Wang C, Hou X, Snyder PJ (2017). Testosterone treatment and cognitive function in older men with low testosterone and age-associated memory impairment. JAMA.

[5] Budoff MJ, Ellenberg SS, Lewis CE, Mohler ER, Wenger NK, Bhasin S, Barrett-Connor E, Swerdloff RS, Stephens-Shields A, Cauley JA, Crandall JP, Cunningham GR, Ensrud KE, Gill TM, Matsumoto AM, Molitch ME, Nakanishi R, Nezarat N, Matsumoto S, Hou X, Basaria S, Diem SJ, Wang C, Cifelli D, Snyder PJ (2017). Testosterone treatment and coronary artery plaque volume in older men with low testosterone. JAMA.

[6] Cunningham GR, Stephens-Shields AJ, Rosen RC, Wang C, Bhasin S, Matsumoto AM, Parsons JK, Gill TM, Molitch ME, Farrar JT, Cella D, Barrett-Connor E, Cauley JA, Cifelli D, Crandall JP, Ensrud KE, Gallagher L, Zeldow B, Lewis CE, Pahor M, Swerdloff RS, Hou X, Anton S, Basaria S, Diem SJ, Tabatabaie V, Ellenberg SS, Snyder PJ (2016). Testosterone treatment and sexual function in older men with low testosterone levels. J Clin Endocrinol Metab.

[7] Snyder PJ, Bhasin S, Cunningham GR, Matsumoto AM, Stephens-Shields AJ, Cauley JA, Gill TM, Barrett-Connor E, Swerdloff RS, Wang C, Ensrud KE, Lewis CE, Farrar JT, Cella D, Rosen RC, Pahor M, Crandall JP, Molitch ME, Cifelli D, Dougar D, Fluharty L, Resnick SM, Storer TW, Anton S, Basaria S, Diem SJ, Hou X, Mohler ER, Parsons JK, Wenger NK, Zeldow B, Landis JR, Ellenberg SS, Testosterone Trials Investigators (2016). Effects of testosterone treatment in older men. N Engl J Med.

[8] Nobles AL, Leas EC, Althouse BM, Dredze M, Longhurst CA, Smith DM, Ayers JW (2019). Requests for diagnoses of sexually transmitted diseases on a social media platform. JAMA.

[9] fox S, Duggan M (2013). Health Online 2013.

[10] Cassis C, Bassett J (2018). Reddit's year in review.

[11] subreddit r/Testosterone.

[12] Shaban H (2019). Twitter reveals its daily active user numbers for the first time. Washinton Post.

[13] subreddit directory.

[14] Kearney M (2018). rtweet: collecting Twitter data (R package version 0.6.7).

[15] Chung C, Pennebaker J (2008). Revealing dimensions of thinking in open-ended self-descriptions: an automated meaning extraction method for natural language. J Res Pers.

[16] Boyd RL, Pennebaker JW (2015). Did Shakespeare write double falsehood? Identifying individuals by creating psychological signatures with text analysis. Psychol Sci.

[17] Barrett A, Murphy M, Blackburn K (2018). “Playing hooky” health messages: apprehension, impression management, and deception. Health Commun.

[18] Boyd R (2018). MEH: Meaning Extraction Helper (Version 2.1.06).

[19] Blackburn KG, Yilmaz G, Boyd RL (2018). Food for thought: exploring how people think and talk about food online. Appetite.

[20] Jiang T, Osadchiy V, Mills JN, Eleswarapu SV (2020). Is it all in my head? Self-reported psychogenic erectile dysfunction and depression are common among young men seeking advice on social media. Urology.

[21] Pett M, Lackey N, Sullivan J (2003). Making Sense of Factor Analysis.

[22] Wolf M, Chung CK, Kordy H (2010). Inpatient treatment to online aftercare: e-mailing themes as a function of therapeutic outcomes. Psychother Res.

[23] Stanton AM, Boyd RL, Pulverman CS, Meston CM (2015). Determining women's sexual self-schemas through advanced computerized text analysis. Child Abuse Negl.

[24] Kravitz RL (2017). Direct-to-consumer advertising of androgen replacement therapy. JAMA.

[25] Bandari J, Ayyash OM, Emery SL, Wessel CB, Davies BJ (2017). Marketing and testosterone treatment in the USA: a systematic review. Eur Urol Focus.

[26] Balasubramanian A, Thirumavalavan N, Srivatsav A, Yu J, Lipshultz LI, Pastuszak AW (2019). Testosterone imposters: an analysis of popular online testosterone boosting supplements. J Sex Med.

[27] Halpern JA, Brannigan RE (2019). Testosterone deficiency. JAMA.

[28] Gott M, Hinchliff S (2003). Barriers to seeking treatment for sexual problems in primary care: a qualitative study with older people. Fam Pract.

[29] Osadchiy V, Mills JN, Eleswarapu SV (2020). Understanding patient anxieties in the social media era: qualitative analysis and natural language processing of an online male infertility community. J Med Internet Res.

[30] Houman JJ, Eleswarapu SV, Mills JN (2020). Current and future trends in men's health clinics. Transl Androl Urol.

[31] Koch-Weser S, Bradshaw YS, Gualtieri L, Gallagher SS (2010). The Internet as a health information source: findings from the 2007 Health Information National Trends Survey and implications for health communication. J Health Commun.

